# Supplementary material for: Splice-Junction-Based Mapping of Alternative Isoforms in the Human Proteome
Source: Cell Rep. Author manuscript; Available in PMC 2020 Jan 15. (PMC6961840; doi:10.1016/j.celrep.2019.11.026)
Supplement: 2 [file NIHMS1546469-supplement-2.zip › DF1/PXD010154/Liver/Liver_69_PLCD1_NQSSTTAILSLPR.pdf]

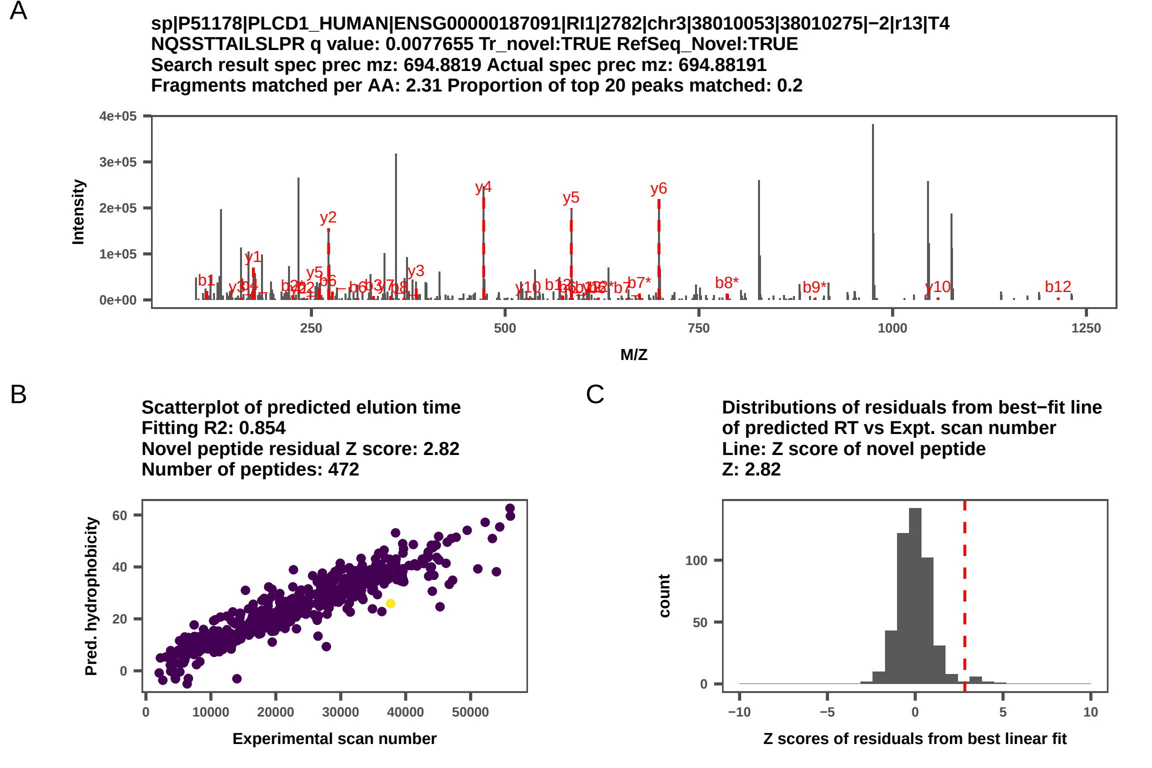
A
sp|P51178|PLCD1_HUMAN|ENSG00000187091|RI1|2782|chr3|38010053|38010275|−2|r13|T4
NQSSTTAILSLPR q value: 0.0077655 Tr_novel:TRUE RefSeq_Novel:TRUE
Search result spec prec mz: 694.8819 Actual spec prec mz: 694.88191
Fragments matched per AA: 2.31 Proportion of top 20 peaks matched: 0.2
Intensity
4e+05
3e+05
2e+05
1e+05
0e+00
M/Z
250
500
750
1000
1250
y1
y2
y3
y4
y5
y6
y10
b1
b7*
b8*
b9*
b12
B
Scatterplot of predicted elution time
Fitting R2: 0.854
Novel peptide residual Z score: 2.82
Number of peptides: 472
Pred. hydrophobicity
60
40
20
0
Experimental scan number
0
10000
20000
30000
40000
50000
C
Distributions of residuals from best−fit line
of predicted RT vs Expt. scan number
Line: Z score of novel peptide
Z: 2.82
count
100
50
0
Z scores of residuals from best linear fit
−10
−5
0
5
10
